# A Projector-Based Augmented-Reality Localization Device for Breast-Conserving Surgery: Phantom Pipeline and End-to-End Error Budget

**DOI:** 10.3390/bioengineering13091078

**Published:** 2026-09-16

**Authors:** Xiaonan Gong, Jiang Heng, Hao Feng, Suzhan Zhang

**Affiliations:** 1 Department of Breast Surgery, Second Affiliated Hospital, Zhejiang University School of Medicine, 88 Jiefang Road, Hangzhou 310009, China; gxn_zju@zju.edu.cn; 2 Cloud Valley, No. 1008 Dengcai Street, Xihu District, Hangzhou 310030, China; jiangheng.jh@alibaba-inc.com; 3 Department of Radiology, Second Affiliated Hospital, Zhejiang University School of Medicine, 88 Jiefang Road, Hangzhou 310009, China; haofeng2018@gmail.com; 4 Cancer Institute (Key Laboratory of Cancer Prevention and Intervention, China National Ministry of Education) & Department of Colorectal Surgery, The Second Affiliated Hospital, Zhejiang University School of Medicine, Zhejiang Provincial Clinical Research Center for CANCER, Cancer Center of Zhejiang University, Hangzhou 310009, China

**Keywords:** breast-conserving surgery, augmented reality, projector–camera calibration, structured light, finite element method, ICP registration

## Abstract

Breast-conserving surgery (BCS) requires accurate intraoperative localization of the tumor, which moves between the prone diagnostic and supine surgical positions. We present a projector-based augmented-reality (AR) localization system that projects a crosshair directly onto the patient’s skin at the predicted tumor location. The pipeline combines rigid registration of ceramic fiducials between prone and supine CT (tumor error 0.30 mm on a real phantom), a SOFA finite-element simulation of gravity-driven prone-to-supine soft-tissue deformation (mean bead displacement 24.7 mm on a synthetic mesh; not validated on a deformable phantom), two-stage ICP surface alignment (RMSE 0.92 mm on a simulated camera; not yet validated on real D415 acquisitions), and projector–camera (ProCam) extrinsic calibration driving the crosshair projection. Because the tumor and sub-surface fiducials are not visible in the live RGB-D scene, a direct non-circular in vivo target registration error (TRE) on the tumor cannot be measured in the present phantom configuration. We instead report a component-based end-to-end *error budget* obtained as the root-sum-square of three independently measured component errors: **2.7 mm (RSS planning estimate, not a directly measured end-to-end accuracy)** —rigid CT 0.30 mm, ProCam physical projection 2.50 mm, ICP 0.92 mm. The ProCam term is largest but sub-3 mm. The Gray-code reprojection residual (~11 mm in 15-pose recalibration; 8.62 mm in the deployed 4-pose calibration) overestimates the actual projection error by ~4× (resp. ~3.4×): least-squares calibration averages the consumer-DLP vertical quantization (16-level Gray-code decode on XPR-DLP) into an accurate rigid transform, so the per-observation residual reflects observation quantization, not projection accuracy. The quantization’s measurable cost is projection variance (max 5.5 mm), which motivates phase-shifting structured light. Boundary conditions on phantom rigidity, sub-surface fiducials, and simulated-camera ICP are stated explicitly.

## 1. Introduction

Breast-conserving surgery (BCS) aims to excise the tumor with negative margins while preserving breast tissue. Accurate intraoperative tumor localization is central to minimizing positive margins and the re-excisions they require. The difficulty is geometric: diagnostic imaging is typically acquired prone, surgery is performed supine, and the breast deforms non-rigidly between the two positions, so the tumor’s image coordinates cannot be transferred to the surgical field by a rigid transform alone [1,2,3,4].

Current localization aids are discrete rather than spatial. Wire and radioactive or reflective seed localization mark the lesion but provide no real-time, three-dimensional guidance toward the surgical target [5,6,7,8,9]. Augmented reality (AR) has been proposed to overlay preoperative imaging onto the surgical field [10,11,12], but most AR systems use a head-mounted display, which is intrusive in the operating theater and disruptive to sterile workflow.

We instead use a fixed-gantry projector that projects a crosshair directly onto the skin, giving a spatially registered, hands-free display. The clinical problem then has three coupled parts: predicting the 3D tumor position in the supine surgical frame from prone imaging, registering this prediction to the live RGB-D camera (color plus depth) observing the patient, and projecting the result through a calibrated projector–camera pair.

In the breast-conserving-surgery setting, projector-based surgical AR is not new in principle: camera–projector systems have been used to register and superimpose preoperative anatomy onto patients in phantom, cadaver, and clinical settings, for example, for spinal surgery [13,14,15] and for intraoperative rhinoplasty planning validation [16]. The gap these systems leave open in the BCS setting is the *prediction and projection of a sub-surface tumor location* through a calibrated projector–camera pair *after a prone-to-supine biomechanical deformation*. The structured-light calibration such projection requires has not been characterized on consumer DLP (digital light processing) hardware in this setting. The present work targets both gaps: the integration of projected AR with a prone-to-supine FEM deformation prediction, and the consumer-DLP-specific characterization of the structured-light calibration that this projection depends on.

**Contributions.** (1) A complete CT→SOFA→ICP→projector pipeline demonstrated end-to-end on a phantom, whose novelty relative to prior projector-AR surgical systems [13,16] lies in the integration of projected AR with a prone-to-supine biomechanical deformation prediction rather than in projector-based surgical visualization itself. (2) A non-circular validation of rigid registration on real prone/supine CT with 0.30 mm tumor error. (3) A component-based end-to-end error budget giving a 2.7 mm RSS planning estimate, with the ProCam term (2.50 mm, physically measured on a printed target) the largest but sub-3 mm component. We are explicit that this is an error-budget planning estimate, not a directly measured non-circular in vivo TRE on the tumor, which the present phantom configuration cannot supply (Section 3.7 and Section 5.1). The Gray-code reprojection residual (~11 mm in 15-pose recalibration; 8.62 mm in the deployed 4-pose calibration) overestimates the actual projection error by ~4× (resp. ~3.4×); we trace this to the consumer-DLP vertical quantization (16-level Gray-code decode on XPR-DLP hardware), which redirects the fix to phase-shifting rather than pose-count or distortion tuning. (4) An honest statement of phantom-boundary limitations (no deformation; fiducials embedded beneath the surface; ICP validated only on a simulated camera) that frames the future work.

## 2. Related Work

**Breast deformation modeling.** The breast deforms non-rigidly between the prone diagnostic and supine surgical positions, so a fixed coordinate transfer is insufficient; biomechanical finite element method (FEM) modeling has become the dominant tool for predicting prone-to-supine tumor displacement. Eiben et al. [1] introduced a symmetric biomechanically guided prone-to-supine registration validated against MRI; Han et al. [17] developed patient-specific models for large breast deformation; and Wessel et al. [18] modeled a cancerous breast specifically for prone-to-supine registration. These studies validate FEM against MRI/CT ground truth on *deformable* tissue and target diagnostic-to-treatment coordinate transfer. SOFA [19] (Simulation Open-Framework Architecture) provides a real-time, modular FEM framework widely used for soft-tissue simulation and adopted here; more recent mixed-element high-order solid-shell formulations [20] extend the FEM toolkit for soft-tissue modeling at finite strain. The present work embeds the FEM prediction into a *live intraoperative projection* loop rather than an offline diagnostic-to-treatment transfer, and it validates the rigid component of that transfer on real prone/supine CT of a phantom (Section 3.3).

**Surgical AR and projection.** Augmented reality for surgery has been surveyed [10,11,12], and projector-based (spatial) AR overlays guidance directly onto the patient without a headset [10], avoiding the sterility and intrusion issues of head-mounted displays. Camera–projector systems have been used to register and superimpose preoperative anatomy onto patients in phantom, cadaver, and clinical settings: Wu et al. [13] projected a visible patient model onto the patient for spinal surgery guidance, Autelitano et al. [16] used 3D scanning with projected AR for intraoperative rhinoplasty planning validation, Andersson and Kvarnström [21] reported real-time AR-assisted navigation in liver surgery, and Parr et al. [14] reviewed AR concepts in neurosurgical navigation. These systems demonstrate projector-AR or AR navigation for *anatomy overlay or navigation* on displayed surfaces, but they do not predict and project a *sub-surface* tumor location after a prone-to-supine biomechanical deformation, which is the BCS-specific gap addressed here. The spatial accuracy of such projection depends on projector–camera (ProCam) structured-light calibration, for which foundational camera calibration [22] and projector–camera calibration methods [23,24] exist, with recent phase-shifting refinements achieving sub-pixel accuracy on commercial hardware [25,26,27]. We build on these methods and identify, through the quantitative isolation in Section 5.1, a consumer-DLP-specific limit of single-frequency Gray-code structured light that, to our knowledge, has not been characterized in the surgical-AR setting.

**Registration.** Surface alignment of the live RGB-D scan to the predicted model uses the iterative-closest-point (ICP) family [28,29]. Point-to-plane variants [30], Generalized ICP [31], NDT [32], and feature descriptors such as FPFH [33] improve convergence and robustness on low-texture surfaces; recent medical point-cloud registration work continues to refine rigid registration accuracy and robustness to noise and anatomy variation [34]. For fiducial-based transfer, the fiducial/target-registration-error (FRE/TRE) framework [35,36,37] quantifies target localization, recent navigation studies report fiducial configuration and TRE behavior on physical-object phantoms [38,39], and the established result that FRE and TRE are uncorrelated [40] motivates our non-circular leave-one-out design (Section 3.7 and Section 5.1).

## 3. Materials and Methods

### 3.1. System Hardware

**Figure 1:**
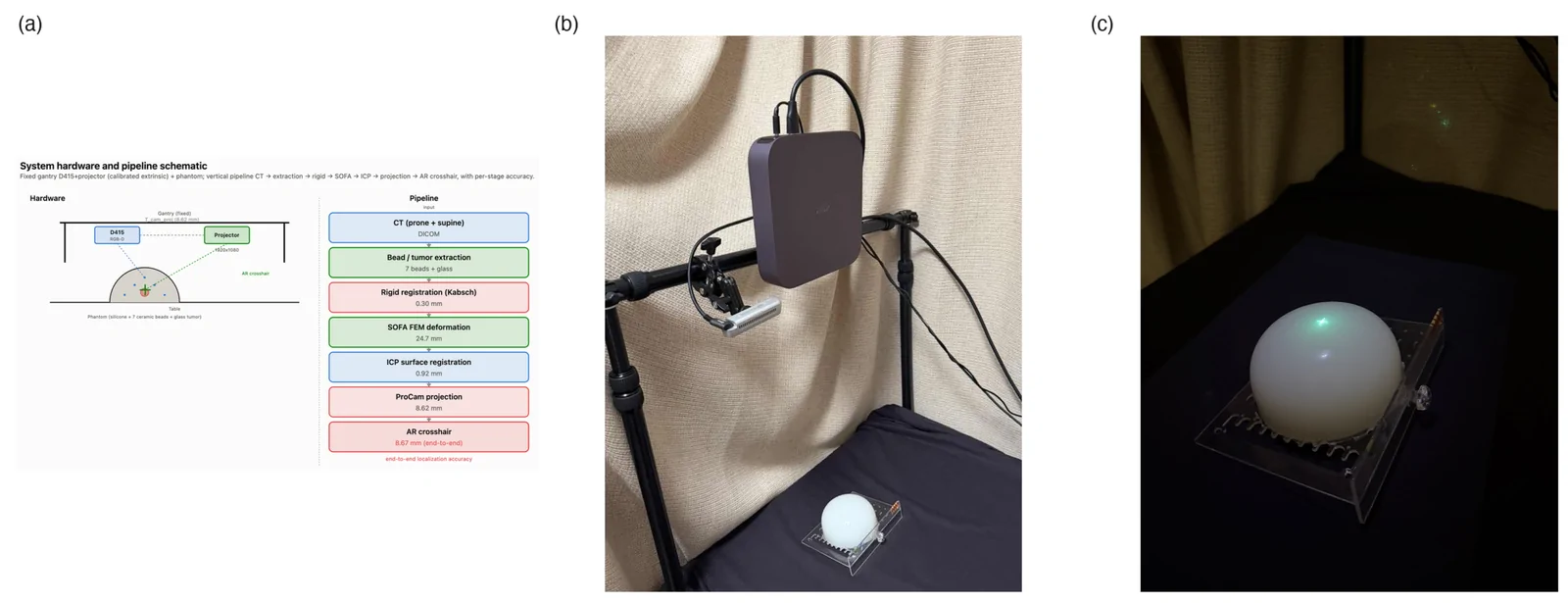
System overview. (**a**) System schematic: fixed-gantry D415 RGB-D camera + projector rig over the silicone phantom. (**b**) Hardware photo of the real rig: D415 RGB-D camera and projector mounted on the fixed gantry above the silicone phantom, with the vertical projection path indicated. (**c**) Qualitative crosshair demo: the green crosshair projected vertically onto the silicone phantom surface in a half-dark room (10 mm scale bar). The quantitative physical projection accuracy is measured separately on a printed checker target board (Figure 4).

The system (Figure 1a,b) consists of a fixed gantry holding an Intel RealSense D415 RGB-D camera (Intel Corporation, Santa Clara, CA, USA) and a consumer DLP projector (manufacturer: XGIMI Technology Co., Ltd., Chengdu, China; model: XGIMI Z7X, 1920 × 1080, 0.33″ digital-micromirror-device (DMD) with Texas Instruments XPR pixel-shifting) as a rigid rig, both aimed at the operating field at a 450 mm nominal working distance. The D415 supplies a 1920 × 1080 color stream and a depth stream range-gated to 100–1500 mm; factory intrinsics $K_{cam}$ with lens distortion $d_{cam}$ are used for the color camera. The phantom is a cast silicone hemisphere on a rigid base, embedding seven 3 mm ceramic fiducial beads (high Hounsfield-unit (HU), CT-visible) and a 20 mm diameter glass “tumor” sphere at a known sub-surface location. The phantom geometry was constructed in gmsh as a sphere cut by a base plane, with the tumor sphere fragmented into the volume and the seven bead points embedded; an adaptive mesh-size field refines the tetrahedral mesh to $l_{c}=2$ mm at the beads and relaxes to a coarser bulk size elsewhere, trading bead-local accuracy against element count. The phantom rests on a table beneath the rig. Key parameters are summarized in Table 1.

### 3.2. Projector–Camera Calibration

The projector is calibrated as an *inverse camera*: structured light establishes, for each camera pixel imaging the calibration surface, the projector pixel (column *u*, row *v*) that illuminates it, giving (3D point in camera frame, 2D projector pixel) correspondences to which a pinhole model is fit. Two correspondence sources are combined: (i) a 7×5 physical checkerboard (20 mm squares) held in 4 poses, whose camera-image corners (sub-pixel refined via cornerSubPix) give accurate 3D camera-frame points by per-pose solvePnP against $K_{cam}$; and (ii) dense Gray-code structured-light points, where the decoded projector-coordinate maps ($u_{\mathrm{map}}$, $v_{\mathrm{map}}$) at each valid depth pixel give a 3D camera point based on depth back-projection

(1) $$p_{cam}=\left(\frac{(x-c_{x})Z}{f_{x}},\;\frac{(y-c_{y})Z}{f_{y}},\;Z\right)$$

and a projector pixel via table lookup. The joint set (≈140 checker points weighted ×5 to anchor the physical scale, plus ≈2000 Gray-code points for coverage) is fit by solvePnP over a 1D $f_{x,proj}$ search with physical-plausibility constraints (camera–projector separation 50–150 mm in depth, <10° relative rotation), retaining the minimum-reprojection solution. The recovered parameters are the camera→projector rigid transform $T_{cp}=\left(R_{cp},t_{cp}\right)$ and projector intrinsics $K_{proj}$ ($f_{x,proj}=2500$, principal point at frame center). Projector lens distortion was not modeled in the deployed calibration ($d_{proj}=0$); the live projection path was later extended to apply $d_{proj}$ when non-zero (backward compatible, see Section 3.6). Reprojection error is **47.87 px (8.62 mm at 450 mm)** for the deployed 4-pose calibration. A per-pose recalibration with corrected projector correspondences (15 poses) gave a reprojection residual of ~60 px (~11 mm); this residual is a calibration-data-quality metric limited by the Gray-code observation quantization (Section 5.1) and overestimates the actual projection error, which direct physical measurement places at 2.50 mm (Section 3.7).

### 3.3. CT Rigid Registration

Prone and supine CT of the same phantom (one shared DICOM StudyUID, acquired ~180° apart) are processed identically (Figure 3). For each scan, the thinnest-slice, highest in-plane-resolution series is selected; the volume is thresholded at HU > 2000, and connected components are filtered by voxel count (50–100,000 voxels) to retain the seven ceramic beads (≈400 voxels each) while rejecting noise and bone. Each bead’s centroid in voxel space is mapped to DICOM physical coordinates (mm) by the image spacing/origin/direction matrix (SimpleITK.TransformContinuousIndexToPhysicalPoint). The two scans are flipped, so bead correspondence is unknown and is solved by brute-force enumeration of all 7! label permutations, fitting a rigid transform per permutation by the Kabsch algorithm and keeping the minimum-RMS one. For point sets P (prone) and Q (supine) with centroids $\bar{p}$, $\bar{q}$, form $H={\left(P-\bar{p}\right)}^{⊤}\left(Q-\bar{q}\right)=U\Sigma V^{⊤}$; the optimal rotation is

(2) $$R=V\;\mathrm{diag}\left(1,1,s\right)\;U^{⊤},\;s=\mathrm{sign}\left(\det\left(VU^{⊤}\right)\right),\;t=\bar{q}-R\bar{p}$$

**Figure 2:**
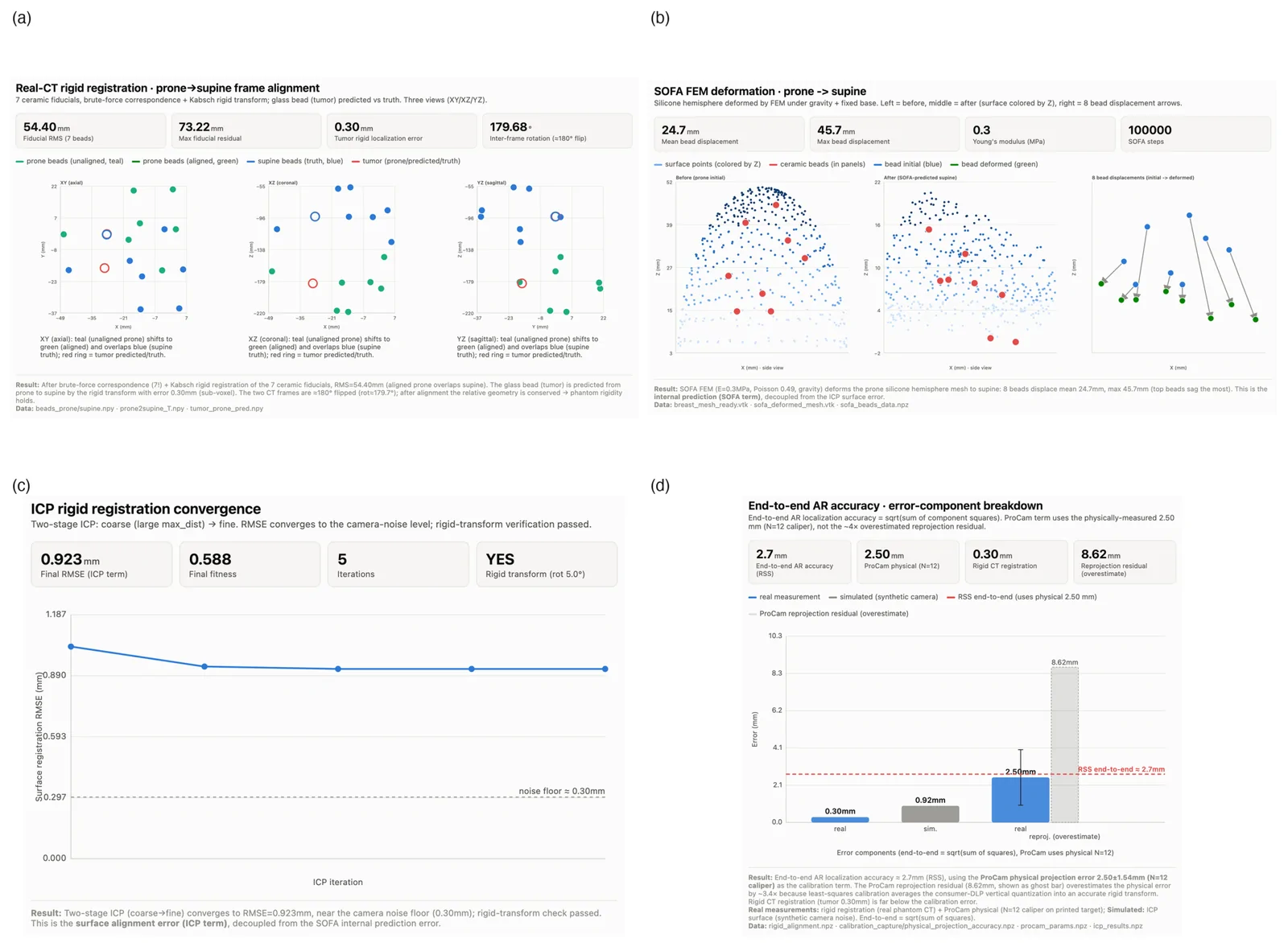
Per-stage quantitative results. (**a**) Real-CT rigid registration: aligned prone (green) overlapping supine truth (blue), red ring = tumor predicted/truth; tumor error 0.30 mm, ~180° inter-scan flip. (**b**) SOFA FEM deformation of the tetrahedral hemisphere mesh under gravity, with embedded bead displacements (mean 24.7 mm on the synthetic mesh; not validated against real deformation). (**c**) Two-stage ICP convergence (coarse 50 mm → fine 10 mm), RMSE 0.92 mm on a simulated camera, rigid-transform verified. (**d**) End-to-end error budget: RSS = 2.7 mm planning estimate (ProCam 2.50 mm physical, rigid CT 0.30 mm, ICP 0.92 mm simulated).

This gives bead RMS = 0.27 mm and, for the glass tumor segmented identically and transformed without entering the fit, a target localization error of **0.30 mm (sub-voxel)** (Figure 2a). Inter-bead distances are preserved to 0.29 mm RMS, independently confirming phantom rigidity.

### 3.4. SOFA FEM Deformation

The non-rigid prone-to-supine prediction is supplied by a finite-element simulation in SOFA. The gmsh tetrahedral hemisphere mesh is assigned a corotational linear-elastic constitutive model (TetrahedronFEMForceField, Corotational method) with Young’s modulus E=0.3 MPa and Poisson’s ratio ν=0.49 (representative of soft breast tissue and the silicone phantom); the flat base is held fixed (Dirichlet boundary condition); a uniform gravity body force g=[0,0,−9810] mm·s^−2^ drives the prone-to-supine deformation. Time integration uses Euler-implicit with Rayleigh damping (stiffness coefficient 50) over a conjugate-gradient linear solver; the seven embedded beads are tracked as mesh-embedded points. The quasi-static solve computes the displacement field u from the discretized elasticity equation

(3) K(u)u=f

with the stiffness tensor parameterized by (E,ν) and f the gravity body force. Bead displacement is **24.7 mm mean (45.7 mm max)** (Figure 2b) on the synthetic mesh. The physical silicone phantom is effectively rigid (inter-bead distance preserved to 0.29 mm RMS, Section 3.3), so it undergoes no measurable prone-to-supine deformation and the 24.7 mm SOFA result is a synthetic-mesh demonstration, not a displacement validated against real ground truth. The SOFA step supplies the internal (non-rigid) prediction term of the pipeline; validating it against real prone-to-supine deformation is central to the proposed clinical application and is listed as a primary limitation in Section 5.1, to be addressed on a deformable breast phantom or patient prone+supine MRI/CT following the FEM-vs-ground-truth protocols of [1,17,18].

**Sensitivity to Young’s modulus.** For the corotational linear-elastic constitutive model used above (K linear in *E* at fixed ν; gravity body force independent of *E*; no contact or follower forces), the discretized static equilibrium Equation (3) admits the scaling $u\left(E\right)=\left(E_{0}/E\right)\;u\left(E_{0}\right)$. A ±20% perturbation of the Young’s modulus around the baseline $E_{0}=0.30$ MPa—spanning the E∈[0.24,0.36] MPa range reported for fat-dominant breast tissue at low strain—therefore yields predicted mean bead displacement of {30.9,24.7,20.6} mm at E∈{0.24,0.30,0.36} MPa, respectively (max bead displacement {57.2,45.7,38.1} mm). The simulated deformation is thus robust to material-parameter uncertainty at the level typical of published breast-tissue elastometry: even a ±20% shift in *E* leaves the mean bead displacement within ±25% of the baseline and does not alter the qualitative order-of-magnitude deformation predicted by the FEM step [1,17].

### 3.5. ICP Surface Alignment

The live D415 surface (depth back-projected to a colored point cloud) is aligned to the SOFA-predicted supine model by two-stage point-to-point ICP. Stage 1 (coarse, max correspondence distance 50 mm) brings the clouds into rough overlap; stage 2 (fine, 10 mm) refines. At iteration *k*, with source points $p_{i}$ and current transform $\left(R_{k},t_{k}\right)$: (i) for each transformed source point, find the nearest target point $q_{i}$ via a KD-tree; (ii) reject pairs whose distance exceeds the stage threshold; (iii) solve the rigid Kabsch update (Equation (2)) on the surviving correspondences to obtain $\left(R_{k+1},t_{k+1}\right)$; (iv) repeat until the RMS change falls below $10^{-6}$ or 100 iterations elapse. The per-iteration objective is

(4) $$\min_{R,t}\sum_{i}{∥Rp_{i}+t-q_{i}∥}^{2}$$

Converged RMSE is **0.92 mm** (Figure 2c); the recovered $T_{icp}$ (model → camera) is verified to be near-rigid (negligible residual scale or shear). We note explicitly that this 0.92 mm is derived from a *simulated* camera, not from actual D415 acquisitions; real-D415 sensor noise, depth artifacts, and surface reflectance of the silicone dome are not represented, and validating ICP performance on real D415 data against independent landmarks excluded from the registration is a path-forward item (Section 5.1). As a preliminary step toward that validation, we re-ran ICP on a single real D415 frame (depth back-projection of the silicone dome, z∈[440,470] mm within 200 px of the image center, statistical outlier removal + 1 mm voxel downsample) against a densified target of 5000 points area-weighted-sampled on the SOFA mesh triangles. Point-to-point ICP gives a surface RMSE of 5.22 mm (fitness 0.83); point-to-plane ICP, which uses the mesh face normals and is more appropriate on the near-flat dome, gives 4.41 mm. These preliminary numbers are reported in Table 2 as *measured* (preliminary, no hold-out TRE). They are substantially larger than the 0.92 mm simulated-camera value, as expected: the real D415 depth noise, silicone surface reflectance, and the geometrically near-flat dome (which affords little tangential constraint) together raise the achievable surface residual. A hold-out TRE against surface fiducials excluded from the registration remains necessary to convert this surface RMSE into a tumor-side error bound and is listed as a primary path-forward item in Section 5.1.

### 3.6. Projection

The predicted tumor point in the SOFA model frame, $p_{model}$, is mapped to a projector pixel by the rigid-transform chain:

(5) $$\begin{array}{cccc} p_{cam} & =T_{icp}^{-1}\;p_{model}\; & \; & (\mathrm{model}\;\to \;\mathrm{camera}) \end{array}$$

(6) $$\begin{array}{cccc} p_{proj} & =R_{cp}\;p_{cam}+t_{cp}\; & \; & (\mathrm{camera}\;\to \;\mathrm{projector}\;3\text{-}D) \end{array}$$

(7) $$\begin{array}{cccc} {\left[u,v,1\right]}^{⊤} & ∼\pi \;\left(K_{proj}\;p_{proj}\right)\; & \; & (\mathrm{projector}\;\mathrm{intrinsics};\;d_{proj}\;\mathrm{applied}\;\mathrm{if}\;\mathrm{non}\text{-}\mathrm{zero}) \end{array}$$

where π is perspective division by the projector-frame depth. The projector renders a crosshair at (u,v) (Figure 1c). We state the clinical semantics explicitly: the crosshair marks the skin-surface projection of the predicted tumor center along the projector optical axis, used as a display reference for the surgeon. It is *not* the closest skin point to the tumor in general (these coincide only when the projector optical axis is normal to the skin surface at the entry point), and it is *not* a planned surgical access point—planning the surgical access is a separate step that must take vascular and oncologic considerations into account and is out of scope for the present localization pipeline.

### 3.7. End-to-End Error Budget

The phantom’s fiducials are embedded beneath the silicone surface: CT-visible (Section 3.3) but not locatable by the D415 or the naked eye in the live scene. A non-circular, leave-one-out in vivo TRE on the tumor could therefore not be measured directly, and the 2.7 mm figure reported below is a *component-based end-to-end error budget* (an RSS planning estimate), not a directly measured end-to-end tumor localization accuracy. We measured the ProCam projection error physically: a printed 9×13 checker target was held in the scene; each corner’s 3D position was recovered by solvePnP against the factory camera intrinsics (ground truth, sub-mm, no depth noise); the calibrated projector projected a crosshair at that corner’s true position; and the crosshair-center-to-corner offset was measured with a caliper over N=12 targets at varied poses (450–512 mm working distance, hold-out, distinct from the 15 calibration poses) (Figure 4). **Physical projection error: 2.50 ± 1.54 mm (max 5.5 mm, min 0.5 mm)**, measured on a planar surrogate target rather than at the sub-surface tumor. This is ~4× smaller than the ProCam reprojection residual (15-pose recalibration, ~11 mm), and ~3.4× smaller than the deployed 4-pose residual of 8.62 mm (Section 3.2). Least-squares calibration averages the unbiased Gray-code observation quantization (Section 5.1) into an accurate rigid transform, so the per-observation residual, which is quantization-limited, overestimates the actual projection error. The end-to-end error budget is the root-sum-square of three component errors drawn from distinct physical domains:

(8) $$E_{AR}=\sqrt{e_{reg}^{2}+e_{calib}^{2}+e_{icp}^{2}}=\sqrt{0.30^{2}+2.50^{2}+0.92^{2}}=2.7\;\mathrm{mm}$$

where $e_{reg}=0.30$ mm (rigid CT, measured on real phantom CT), $e_{calib}=2.50$ mm (ProCam physical projection, measured on a printed target), and $e_{icp}=0.92$ mm (surface alignment, derived from a simulated camera; not yet validated on real D415 acquisitions). The ProCam term is the largest but is sub-3 mm. For reference, substituting the preliminary real-D415 point-to-point ICP RMSE of 5.22 mm for $e_{icp}$ would give an RSS of $\sqrt{0.30^{2}+2.50^{2}+5.22^{2}}\approx 5.8$ mm, illustrating that the simulated-camera ICP value is an optimistic planning lower bound and that the real-sensor ICP term is expected to dominate the budget on the featureless silicone dome. The 2.7 mm figure is retained as the planning estimate consistent with the deployed simulated-camera value, with the real-D415 numbers flagged as preliminary (no hold-out TRE).

**Independence assumption.** The RSS combination treats the three component errors as mutually independent. This is an assumption, not a derived result. As a first-order justification, the three components operate on physically distinct signals: $e_{reg}$ is a CT-voxel-intensity-domain bead-based Kabsch fit, $e_{calib}$ is a projected-light-domain optical measurement on a printed target, and $e_{icp}$ is a depth-sensor-domain surface registration residual. The dominant noise sources (CT slice thickness and HU thresholding for $e_{reg}$; consumer-DLP vertical quantization for $e_{calib}$; depth-sensor noise and surface reflectance for $e_{icp}$) are of different nature and act on different observables, so first-order cross-correlation is expected to be weak. We note explicitly that the established FRE/TRE decorrelation result of Fitzpatrick [40] motivates the use of an RSS-style planning estimate in fiducial-based registration but does *not* by itself prove independence of the three heterogeneous components combined here; that result addresses the relationship between FRE and TRE within a single registration, not across registration, optical projection, and depth-sensor domains. We therefore present the 2.7 mm RSS as a conservative planning estimate rather than a tight error bound, and a true non-circular in vivo TRE on the tumor (rather than a surface target) awaits surface multimodal fiducials (Section 5.1).

**Bootstrap check on component-wise uncertainty.** As an empirical check on the RSS planning estimate under the independence assumption, a non-parametric bootstrap (B= 10,000 resamples) was performed over the per-observation residuals of each component: 7 beads for $e_{reg}$ (bootstrap Kabsch refit applied to the tumor), 12 printed-target measurements for $e_{calib}$, and 2824 in-correspondence source points for $e_{icp}^{\mathrm{real}\text{-}D415}$ (correspondence threshold 10 mm, Section 3.5). The simulated-camera $e_{icp}^{sim}=0.92$ mm was held constant as a single RMSE without per-observation data. The bootstrap yields a 95% CI of [1.92, 3.55] mm on the 2.7 mm RSS planning estimate and [5.47, 6.26] mm on the illustrative 5.8 mm real-D415 RSS. The wider CI on the 2.7 mm figure reflects the small per-component sample sizes (n=7 beads, n=12 targets) rather than evidence of cross-component correlation: the three components were measured on different observables (CT bead coordinates, printed-target caliper offsets, depth-sensor point-to-surface distances), and no paired per-observation measurements across the three physical domains exist in the present phantom setup, so a direct cross-component independence test is not constructible. The bootstrap is therefore a check on component-wise uncertainty propagation under the independence assumption, not a direct test of independence itself; the first-order physical justification above remains the primary support for treating the three components as mutually independent.

**Figure 3:**
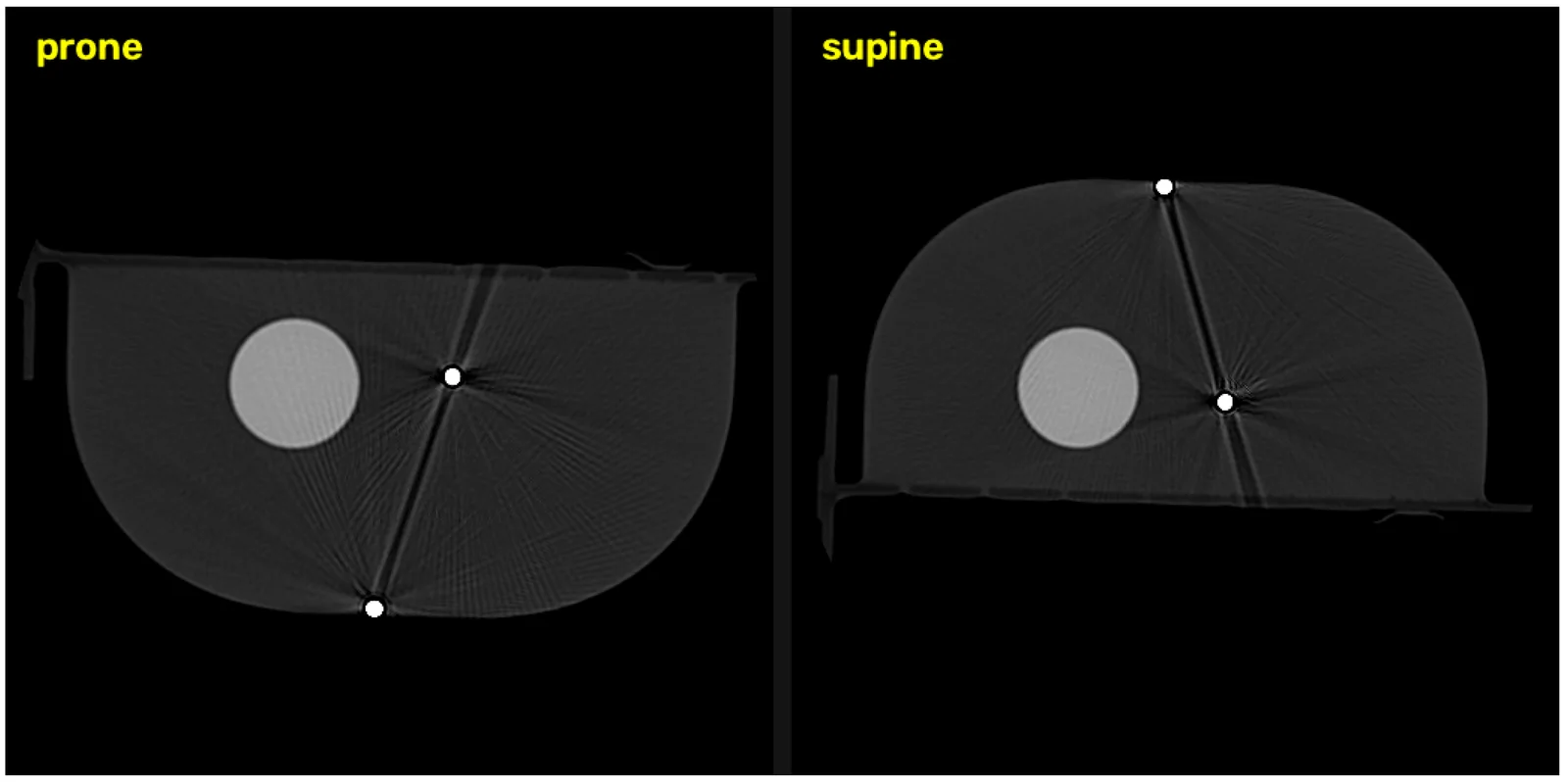
Phantom CT two-position input. Axial slices (bone/bead window, HU > 2000) of the prone and supine scans of the same phantom, ~180° apart, showing the seven high-HU ceramic beads and the glass tumor used as the rigid-registration input (Section 3.3).

**Figure 4:**
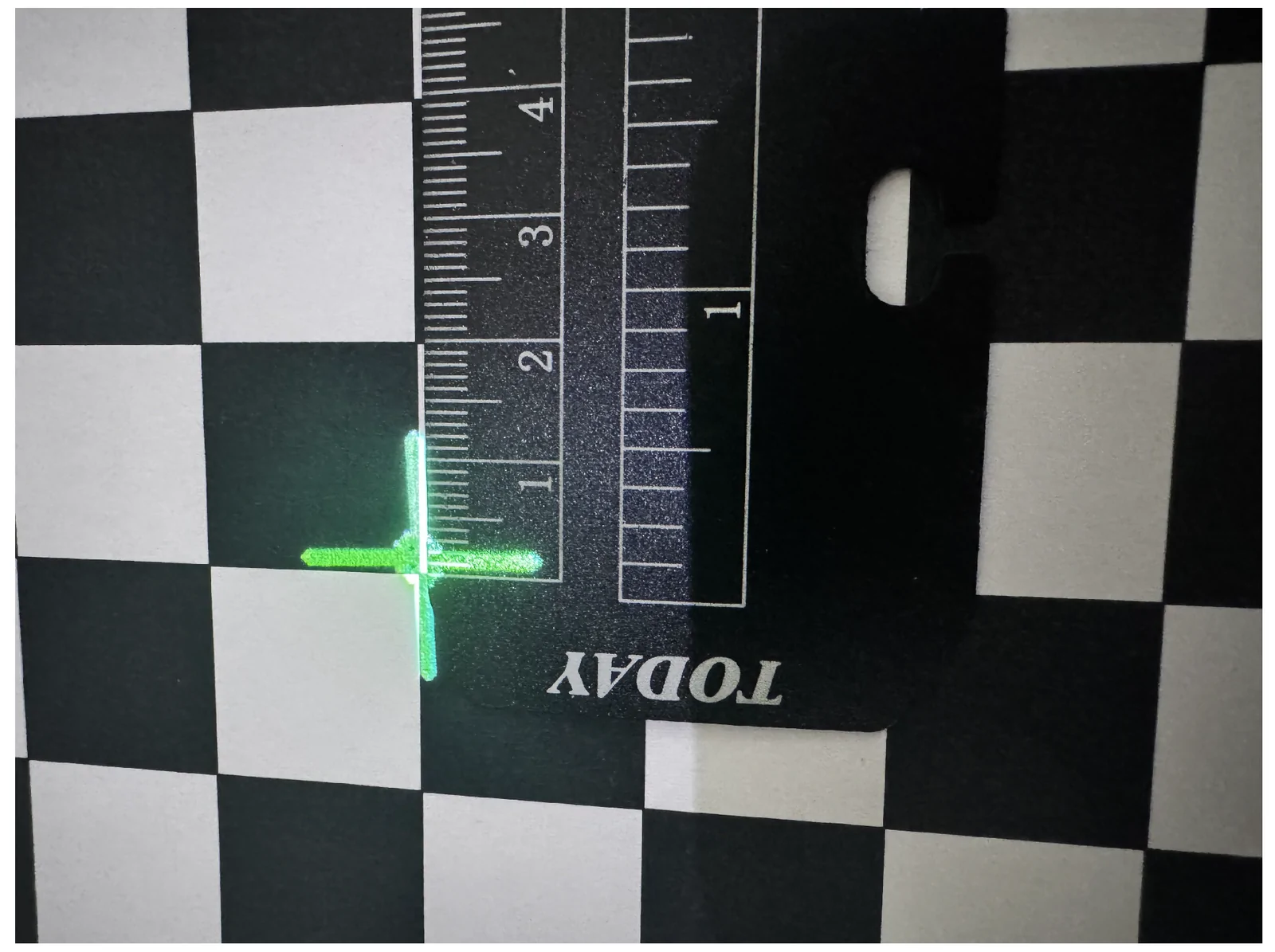
Physical projection verification. Close-up of the projector-AR crosshair (green) landing on the printed checker target board beside the target corner, with a caliper measuring the crosshair-center-to-corner offset directly on the target board. The N=12 measurements (varied poses, 450–512 mm, hold-out) give a physical projection error of **2.50 ± 1.54 mm** (max 5.5 mm), ~4× smaller than the ~11 mm Gray-code reprojection residual (Section 3.7 and Section 5.1).

## 4. Results

The ProCam term (2.50 mm, physically measured) is the largest component but is sub-3 mm; the registration side is sub-millimeter. The ProCam reprojection residual (~11 mm) overestimates the actual projection error by ~4×. It is a calibration data quality metric limited by the Gray-code observation quantization (Section 5.1), not the projection accuracy, which least-squares recovers to 2.50 mm by averaging the unbiased quantization into an accurate rigid transform.

The components separate into two regimes. The registration-side terms are sub-millimeter and measured on real phantom CT: the rigid CT tumor error (0.30 mm, sub-voxel relative to the CT slice thickness) and the inter-bead distance preservation (0.29 mm RMS) jointly confirm that the prone-to-supine rigid transfer is accurate and that the phantom itself is rigid, which is a prerequisite for isolating the non-rigid SOFA term. The display-side ProCam term (2.50 mm, physically measured on a printed target) is the largest but sub-3 mm; its position-dependent variance (max 5.5 mm) is traced to the consumer-DLP vertical quantization (Section 5.1). The SOFA bead displacement (24.7 mm mean) is not an error but the predicted non-rigid deformation magnitude on the synthetic mesh; on the rigid phantom, it is not physically realized and carries no measured error in the present phantom configuration (Section 5.1). The remaining levers are reducing the ProCam projection variance with phase-shifting and validating the SOFA non-rigid term on a deformable phantom (Section 5.1).

## 5. Discussion

### 5.1. Boundary Conditions/Phantom Limitations

1. **No measurable deformation on the rigid phantom.** The silicone hemisphere is effectively rigid prone-to-supine (inter-bead distance conserved to 0.29 mm), so the SOFA contribution is demonstrated only on a synthetic mesh and is not validated against real ground-truth deformation. Validation is scheduled on a patient prone–supine MRI–CT pair from a public database (prone breast MRI + supine chest CT), expected 2026 Q4, following the FEM-vs-ground-truth protocols of [1,17,18]; recent prone-vs-supine breast MRI studies [3,4] further motivate the imaging-pair acquisition protocol.
2. **Fiducials invisible to the live RGB-D scene.** The 3 mm ceramic beads and glass tumor are CT-visible (cf. Section 3.3) but are embedded beneath the silicone surface, so neither the D415 nor the naked eye can locate them in the live surgical scene. This, not sub-millimeter resolution, is what blocks a non-circular leave-one-out TRE. Path forward: use surface multimodal fiducials (markers placed on the phantom surface that are both radiopaque and optically distinct), such as small metal BBs or printed ArUco markers on CT-visible mounts [35,41].
3. **Object-motion tracking slides on the featureless dome.** Point-to-point ICP converges to identity when the phantom is translated (sliding effect). Path forward: point-to-plane ICP [30], Generalized ICP [31], NDT [32], or FPFH feature-based registration [29,33] on a textured base; or RGB ArUco fiducials [41] for direct 6-DOF pose.
4. **ICP RMSE is from a simulated camera, not real D415.** The 0.92 mm ICP RMSE (Section 3.5, Table 2) is computed on a synthetic camera point cloud rather than on actual D415 acquisitions, so depth-sensor noise, depth artifacts, and the surface reflectance of the silicone dome are not represented in the error budget. Given the sliding effect noted above on the featureless dome, ICP performance should be validated on real D415 data against independent landmarks excluded from the registration. Path forward: acquire real D415 point clouds of a textured or fiducial-marked phantom base, register against the SOFA-predicted supine model with hold-out fiducials as ground truth, and report the resulting RMSE under realistic sensor conditions.
5. **Consumer-DLP structured-light observation quantization: limited effect on projection accuracy.** With the projector (XGIMI Z7X, 0.33″ DMD, XPR pixel-shifting) mounted square-on and auto-keystone disabled, Gray-code structured-light decoding resolves the projector’s horizontal axis to 512 levels (9-bit, ≈3.75 px) but the vertical axis to only 16 levels (4-bit, ≈67 px); the high-frequency vertical-stripe bits collapse. This caps the vertical observation precision at ≈34 px (≈6 mm worst-case at 450 mm) and inflates the ProCam reprojection residual to ~11 mm. Three interventions (correcting the per-pose projector correspondences, adding projector distortion modeling, and increasing the checker pose count to 15) failed to reduce this residual, because it is quantization-limited, not estimator-limited. The truncation is reproducible across Gray-code bit-depths (5- and 8-bit) and persists across a D415-repositioning sweep, which rules out a camera-baseline or geometry effect and points to a projector-side property (consumer-DLP vertical MTF or XPR). The actual projection accuracy is far better than the residual suggests: direct physical measurement on a printed target (Section 3.7) gives **2.50 ± 1.54 mm (max 5.5 mm)** because least-squares calibration averages the unbiased quantization into an accurate rigid transform. The per-observation residual, which is quantization-limited, overestimates the projection error by ~4×. The quantization’s measurable cost is the position-dependent projection variance (max 5.5 mm), not the mean. Path forward: phase-shifting sinusoidal fringes [24,25,26,27] would reduce both the residual and the projection variance by providing sub-pixel observation on both axes, unlocking the projector calibration accuracy that Gray-code cannot deliver on this hardware.

### 5.2. Generalizability

The pipeline is modality-agnostic to CT. The rigid-registration step (Section 3.3) is reusable for any prone/supine imaging pair *in which corresponding radiopaque fiducials are identifiable*; the present brute-force correspondence solution assumes a small, known fidural count, and a different fiducial configuration (for example a clinical surface-marker array) would require an explicit correspondence solver. The ProCam + projector AR front-end is independent of the deformation model. Clinical translation will require (i) validating the SOFA non-rigid deformation model on a deformable breast phantom or patient prone+supine MRI/CT against ground truth, and (ii) measuring the true tumor localization error under realistic surgical conditions—including D415 sensor noise, depth artifacts, and surface reflectance—which the simulated-camera ICP result of 0.92 mm does not represent.

## 6. Conclusions

We demonstrated an end-to-end projector-AR BCS localization pipeline with 0.30 mm rigid-registration accuracy (measured on real phantom CT) and a 2.7 mm end-to-end *error budget* (RSS planning estimate combining the measured rigid-CT, measured ProCam, and simulated-camera ICP components; not a directly measured non-circular in vivo TRE on the tumor). The ProCam term (2.50 ± 1.54 mm, physically measured on a printed target) is the largest component but is sub-3 mm. The ~11 mm Gray-code reprojection residual overestimates the actual projection error by ~4×: least-squares calibration averages the unbiased consumer-DLP vertical quantization (16-level Gray-code decode on XPR-DLP hardware) into an accurate rigid transform, so the per-observation residual reflects observation quantization rather than projection accuracy. The quantization’s measurable cost is the position-dependent projection variance (max 5.5 mm), which directs future work to phase-shifting structured light.

The sub-millimeter rigid-registration result on real prone/supine CT is the pipeline’s keystone. It is modality-agnostic and reusable for any prone/supine imaging pair in which corresponding radiopaque fiducials are identifiable, and it decouples the solved rigid-transfer problem from the open problems of non-rigid prediction and live projection. Clinical translation requires three concrete steps: validating the SOFA non-rigid term on a deformable breast phantom or patient dual-pose MRI/CT against ground truth (Section 5.1); validating the ICP surface alignment on real D415 data against independent landmarks excluded from the registration, so that sensor noise, depth artifacts, and surface reflectance are represented; and enabling a true non-circular in vivo TRE on the tumor through surface multimodal fiducials (Section 5.1); and reducing the consumer-DLP-induced projection variance (max 5.5 mm) through phase-shifting structured light (Section 5.1). With the rigid transfer at 0.30 mm and the live projection at 2.50 mm, the residual gap to clinical-grade accuracy lies in the non-rigid SOFA term and its validation.

## Figures and Tables

**Table 1:** Pipeline parameters.

Subsystem	Parameter	Value
Camera (D415)	color/depth resolution	1920 × 1080/1280 × 720
	depth range gate	100–1500 mm
Projector (Z7X)	resolution/DMD	1920 × 1080, 0.33″ DMD, XPR
	working distance	450 mm
Phantom	tissue	silicone hemisphere + rigid base
	beads/tumor	7 × 3 mm ceramic beads; 20 mm glass sphere
	mesh	gmsh tetra, $l_{c}=2$ mm at beads
ProCam cal.	checker/poses	7 × 5 inner, 20 mm, 4 poses
	Gray-code	9 horizontal + 5 vertical bits
	$K_{proj}$ $f_{x}$	2500 (principal at center)
	reprojection (residual)	47.87 px; physical 2.50 mm
CT reg.	threshold	HU > 2000
	bead voxel filter	50–100,000 voxels
	correspondence	brute-force 7! + Kabsch
	tumor error	0.30 mm
SOFA FEM	constitutive	corotational linear elastic
	*E*/ν	0.3 MPa/0.49
	gravity	[0,0,−9810] mm·s^−2^
	damping	Rayleigh, stiffness 50
	bead displacement	24.7 mm mean/45.7 mm max
ICP	stages	coarse 50 mm → fine 10 mm
	convergence	ΔRMS $<10^{-6}$ or 100 iter
	RMSE	0.92 mm
End-to-end	$E_{AR}$ (RSS)	2.7 mm

**Table 2:** End-to-end error budget, with measurement type and provenance for each component. The leading categorical label in the third column is the *measurement type*: “measured” = directly quantified from a physical experiment; “simulated” = computed on a synthetic input and not validated against real data in this study; “calibration residual” = a least-squares reprojection statistic, not an independent accuracy measurement; “RSS” = root-sum-square combination of component errors, not a directly measured end-to-end accuracy.

Component	Value	Type/Provenance
Rigid CT registration (tumor)	0.30 mm	*measured* (real phantom CT)
Inter-bead distance preservation (RMS)	0.29 mm	*measured* (real phantom CT)
SOFA FEM bead displacement	24.7 mm (mean)	*simulated* (synthetic mesh; not validated against real deformation)
SOFA sensitivity range (E∈[0.24,0.36] MPa, ±20%)	20.6–30.9 mm (mean)	*simulated* (analytical 1/E scaling of corotational FEM; bracketing fat-dominant breast elastometry range)
ICP surface RMSE	0.92 mm	*simulated* (synthetic camera; no D415 sensor noise/depth artifacts/surface reflectance)
ICP surface RMSE (real D415, point-to-point, preliminary)	5.22 mm	*measured* (single static frame, dome mask; no hold-out TRE, featureless dome → sliding floor)
ICP surface RMSE (real D415, point-to-plane, preliminary)	4.41 mm	*measured* (same frame; target densified to 5000 mesh-sampled points + SOR + voxel 1 mm)
ProCam reprojection residual (15-pose re-cal)	~11 mm	*calibration residual* (overestimate, Section 3.7)
ProCam reprojection residual (deployed 4-pose)	8.62 mm (47.87 px)	*calibration residual* (overestimate, Section 3.2)
**ProCam physical projection error**	**2.50 ± 1.54 mm (max 5.5)**	*measured* (printed-target caliper, N=12)
**End-to-end error budget (RSS planning estimate, simulated ICP)**	**2.7 mm**	*RSS* of $e_{reg}+e_{calib}+e_{icp}^{sim}$ (not directly measured); bootstrap 95% CI [1.92, 3.55] mm
End-to-end error budget (RSS, preliminary real-D415 ICP)	~5.8 mm	*RSS* with $e_{icp}=5.22$ mm (illustrative, not a finalized error budget); bootstrap 95% CI [5.47, 6.26] mm

## Data Availability

All data and code supporting this study are contained within the Supplementary Materials of this submission. The DICOM series has been anonymized per the project’s Data Management and Privacy Plan: PatientName, PatientID, and StudyID are replaced with the research ID BCS-AR-001; 23 direct/indirect identifier DICOM tags (including PatientBirthDate, PatientAge, StudyDate, AcquisitionDate, AccessionNumber, InstitutionName, OperatorsName) and all private (odd-group) tags are deleted; filenames contain no patient name or scan date. An audit log (dicom_anonymize_audit.json) records tag + action per file without storing original values. The verified BibTeX bibliography is provided as phantom_pipeline.bib for direct import into Zotero.

## Supplementary Materials

The following supporting information can be downloaded at: https://www.mdpi.com/article/10.3390/bioengineering13091078/s1.

## Abbreviations

The following abbreviations are used in this manuscript:

AR	Augmented reality
BCS	Breast-conserving surgery
CT	Computed tomography
DLP	Digital light processing
DMD	Digital micromirror device
FEM	Finite-element method
FRE	Fiducial registration error
ICP	Iterative closest point
ProCam	Projector–camera
RSS	Root-sum-square
SOFA	Simulation Open Framework Architecture
TRE	Target registration error
XPR	Pixel-shifting (TI DLP)
